# Large Deformation Mechanisms, Plasticity, and Failure of an Individual Collagen Fibril With Different Mineral Content

**DOI:** 10.1002/jbmr.2705

**Published:** 2016-02-11

**Authors:** Baptiste Depalle, Zhao Qin, Sandra J Shefelbine, Markus J Buehler

**Affiliations:** ^1^ Laboratory for Atomistic and Molecular Mechanics (LAMM), Department of Civil and Environmental EngineeringMassachusetts Institute of TechnologyCambridgeMAUSA; ^2^ Department of Mechanical and Industrial EngineeringNortheastern UniversityBostonMAUSA; ^3^ Center for Computational EngineeringMassachusetts Institute of TechnologyCambridgeMAUSA; ^4^ Center for Materials Science and EngineeringMassachusetts Institute of TechnologyCambridgeMAUSA

**Keywords:** BONE, COLLAGEN, HYDROXYAPATITE, CROSSLINKS, BIOMINERALIZATION, BIOMECHANICS, MOLECULAR MODELING

## Abstract

Mineralized collagen fibrils are composed of tropocollagen molecules and mineral crystals derived from hydroxyapatite to form a composite material that combines optimal properties of both constituents and exhibits incredible strength and toughness. Their complex hierarchical structure allows collagen fibrils to sustain large deformation without breaking. In this study, we report a mesoscale model of a single mineralized collagen fibril using a bottom‐up approach. By conserving the three‐dimensional structure and the entanglement of the molecules, we were able to construct finite‐size fibril models that allowed us to explore the deformation mechanisms which govern their mechanical behavior under large deformation. We investigated the tensile behavior of a single collagen fibril with various intrafibrillar mineral content and found that a mineralized collagen fibril can present up to five different deformation mechanisms to dissipate energy. These mechanisms include molecular uncoiling, molecular stretching, mineral/collagen sliding, molecular slippage, and crystal dissociation. By multiplying its sources of energy dissipation and deformation mechanisms, a collagen fibril can reach impressive strength and toughness. Adding mineral into the collagen fibril can increase its strength up to 10 times and its toughness up to 35 times. Combining crosslinks with mineral makes the fibril stiffer but more brittle. We also found that a mineralized fibril reaches its maximum toughness to density and strength to density ratios for a mineral density of around 30%. This result, in good agreement with experimental observations, attests that bone tissue is optimized mechanically to remain lightweight but maintain strength and toughness. © 2015 The Authors. *Journal of Bone and Mineral Research* published by Wiley Periodicals, Inc. on behalf of American Society for Bone and Mineral Research (ASBMR).

## Introduction

Bone is a complex hierarchical composite made of an organic matrix, filled by a mineral phase and water.^1^, ^2^ Its structure is organized to achieve remarkable mechanical performance.^3^, ^4^ The organic matrix, representing around 25% of bone weight, is constituted of more than 90% of type I collagen molecules that assemble in a quarter‐staggered fashion into thin (20 to 500 nm) and long (∼100 μm) fibrils.^5^, ^6^, ^7^ The remaining 10% of the matrix is composed of noncollagenous proteins. The mineral phase represents on average 65% of hydrated bone weight and is formed of nanosized platelets with dimensions ranging from 1 to 7 nm in thickness, 15 to 200 nm in length, and 10 to 80 nm in width.^2^, ^7^, ^8^, ^9^, ^10^ The mineral crystals have an apatitic structure, but compared to its “ideal” form known as hydroxyapatite [Ca_10_(PO_4_)_6_(OH)_2_], bone apatite can contains various substitutions and vacancies.^11^ The brittle apatite crystals play an essential role in bone's mechanical properties because they bring stiffness, strength, and wear resistance to the much softer but tougher organic matrix.^2^ The composite combines the optimal properties of both constituents to form a remarkably stiff and tough low‐density material.^2^ Although the larger‐scale bone structure varies depending on bone type and species, the mineralized collagen fibril structure is highly conserved among species and represents the universal building block of bone.^12^, ^13^ Collagen–apatite composites are not only the basic building blocks of human bone, but they are also among the most abundant class of biomineralized materials in the animal kingdom.^2^, ^5^

Although bone's structure and its mechanical properties have been well studied, the role of the mineral phase in the mineralized collagen fibrils and the way an individual fibril deforms under external forces is still not well understood. Few experimental techniques have been developed to investigate the mechanical response of individual mineralized collagen fibrils.^13^, ^14^, ^15^, ^16^, ^17^ However, the size and complexity of the structure of mineralized collagen fibrils make it challenging to understand the nanoscale mechanisms governing the fibril mechanics. Other studies based on analytical models have uncovered some key mechanistic features of mineralized tissues where experiments reach their limits, shedding some light on the role of mineral platelets in material strengthening.^13^, ^18^, ^19^, ^20^, ^21^, ^22^ These models help link the nanostructure of the tissue to the organ's mechanical response by integrating some levels of the complex hierarchical structure of bone. However, they cannot be used to explore the relationship between nanostructure, chemical composition, and bone mechanics, specifically in the large deformation regime.

In recent years, molecular simulations have been used to overcome some of the experimental limitations and to explore the mineral's influence on the mechanics of single fibrils. Full atomistic molecular dynamics made it possible to assess the mechanics of collagen molecules and fibrils,^23^, ^24^ mineral crystals,^25^, ^26^ and their interface.^27^, ^28^, ^29^, ^30^ Furthermore, large‐scale models have been developed to investigate the interplay between organic collagen molecules and mineral crystals. However, because of their inherent complexity, such models are limited to the study of only a few molecules. To date, exploring the behavior of a whole and finite‐size fibril using full‐atomistic representation has been out of reach.

To capture a single fibril deformation, coarse‐grained models represent a useful alternative to expensive full‐atomistic calculation. Previous idealized models of nascent collagen fibrils have shown that mineral provides additional strength, stiffness, and ultimate strain to the fibrillar structure.^31^ However, these simplified two‐dimensional models ignore some geometric features of the fibril and do not completely capture the macroscopic deformation mechanisms as exposed in recent experimental work.^14^ Therefore, the mesoscale model of mineralized collagen fibril needs further improvements.

The goal of this study was to explore the deformation mechanisms of a single mineralized collagen fibril under tension as a function of mineral density. To do so, we report a three‐dimensional, coarse‐grained model of a single mineralized collagen fibril, integrating the detailed shape and distribution of the mineral platelets and collagen molecules. By varying the mineral density in the fibril and after performing tensile tests using steered molecular dynamics, we identified key mechanisms associated with large‐scale deformations of a mineralized collagen fibril.

## Materials and Methods

### Fibril model geometry

**Figure 1:**
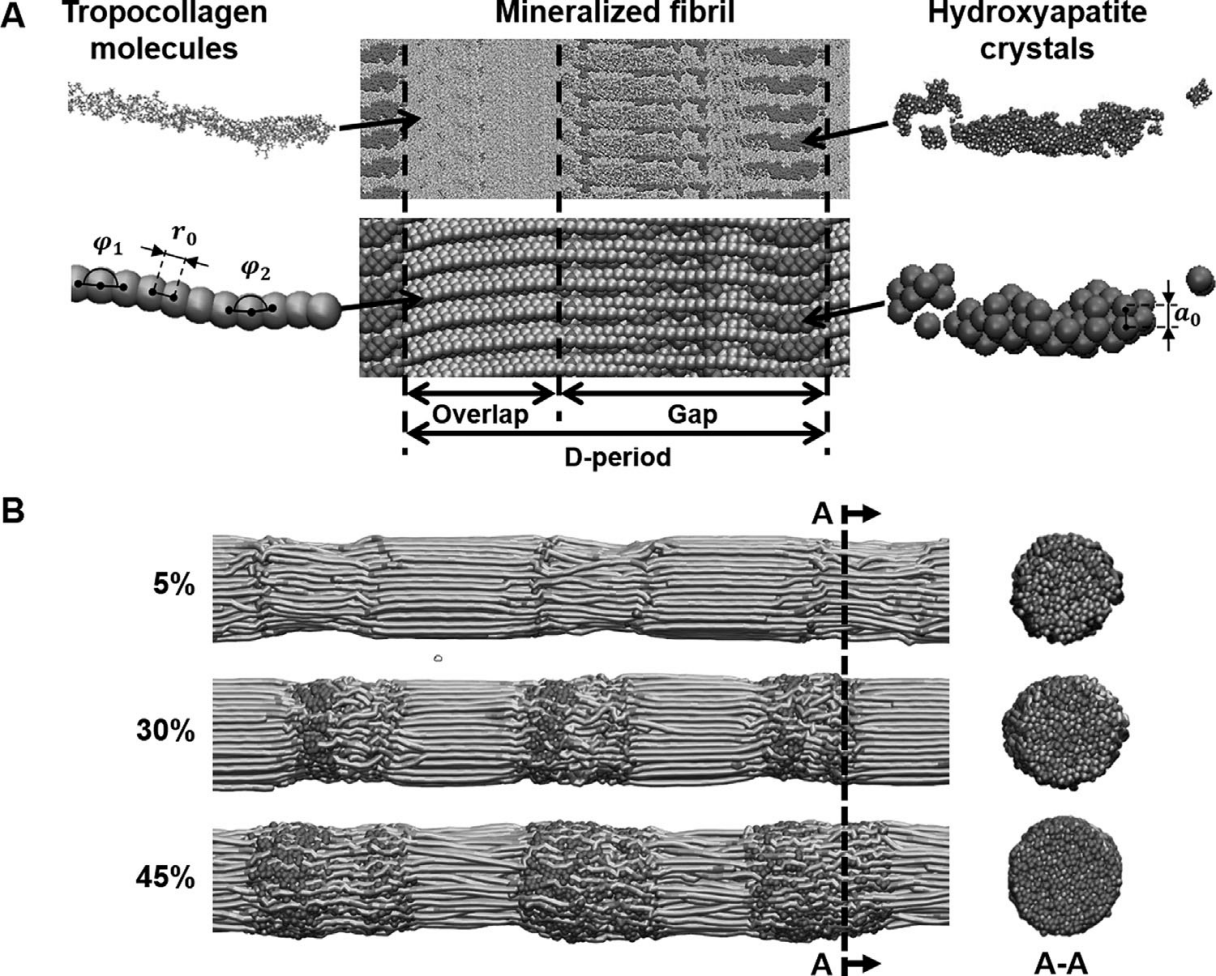
(*A*) Mineralized collagen microfibril full atomistic model (top) and corresponding coarse‐grained model (bottom). The coarse‐grained model retains the main geometric features of the fibril while considerably reducing the number of particles in the model. (*B*) Coarse‐grained model of finite size mineralized collagen fibril with varying mineral density after equilibration. A cross‐section of the fibrils mineralized gap region taken along the dashed line is presented on the right side of the figure.

The geometry of the coarse‐grained models is based on full atomistic simulation of mineralized collagen as previously reported^32^ (Fig. 1 *A*). Further details on the development of the mineralized collagen fibril full atomistic model can be found in Nair and colleagues^32^ and Gautieri and colleagues.^33^ The fibril is built by replication of the tropocollagen molecule and mineral crystal according to the periodicity given by the full atomistic model. The collagen molecules are replicated in order to form a cylinder of diameter *d* = 20 nm to represent a fibril. Only the mineral beads within the fibril are kept (Fig. 1 *B*). Following the in silico mineralization scheme reported in Nair and colleagues,^32^ fibril models with a mineral density ranging from 5% to 45% have been created, with 45% representing the upper limit of the density naturally found in bone. The model of the fibril is built to exhibit five gap/overlap regions along its length, which ensures the periodicity of the model along the axis of the fibril (*x*‐axis). During the simulation, the model is replicated by using periodic boundary conditions along its length to simulate an infinitely long fibril. The length of the model is larger than a single molecule length (∼300 nm) in order to prevent any artifactual interactions between the two ends of a molecule through the periodic boundaries. The resulting model is made up of 158 molecules, which represent a total of 28,305 beads. The number of mineral beads varies between 2037 and 28,962 depending on the mineral's density. We also explore the role of crosslinks in the mineralized fibril mechanics. As observed experimentally, the collagen fibrils naturally display the characteristic staggered arrangement, and highly mineralized fibril tends to lose their diameter periodic variability and display a smoother surface compared to poorly mineralized fibrils (Fig. 1 *B*).

### Coarse‐grain model parameterization

In this study, the interactions between the mesoscopic model particles have been designed according to multi‐body potentials. Further details on the development of the mesoscopic model for solvated collagen molecules can be found in a series of previous publications.^23^, ^34^ Parameters for the mineral phase and collagen‐mineral interaction are developed using a similar approach (see Supporting Information).

The full atomistic representation of both collagen and mineral is simplified and a group of atoms is represented by a single bead (Fig. 1 *A*). This simplification allows us to reach higher time and length scales that are currently not reachable using full atomistic resolution. The total energy of the system is the sum of the energy of the collagen ($E_{collagen}$), the energy of the mineral ($E_{HAP}$), and the energy due to interaction between organic and mineral component ($E_{inter}$):

(1) $$E_{total}=E_{collagen}+E_{HAP}+E_{inter}$$

The energy contribution of the collagen $E_{collagen}$ can be described as:

(2) $$E_{collagen}=E_{bond}+E_{angle}+E_{non-bonded}$$

where $E_{bond}$ represents the energy contribution due to stretching of the collagen molecules, $E_{angle}$ the energy due to bending and $E_{non-bonded}$ the energy due to intermolecular interactions between collagen molecules alone.

A bilinear law matching the nonlinear stress–strain behavior of a single molecule under tension is used to model the stretching energy contributions of collagen.^35^, ^36^ The force between two particles is:

(3) $$F_{bond}\left(r\right)=-\frac{\partial \Phi_{bond}\left(r\right)}{\partial r}$$

where:

(4) $$\frac{\partial \Phi_{bond}\left(r\right)}{\partial r}=\{\begin{array}{ccc} k_{T}^{\left(0\right)}\left(r-r_{0}\right) & \text{if} & r<r_{1}\\ k_{T}^{\left(1\right)}\left(r-r_{0}\right) & \text{if} & r_{1}\leq r<r_{break}\\ 0 & \text{if} & r>r_{break} \end{array}$$

where $k_{T}^{\left(0\right)}$ and $k_{T}^{\left(1\right)}$ are small‐deformation and large‐deformation spring constants. The stretching energy $\Phi_{bond}\left(r\right)$ is given by integrating $F_{bond}\left(r\right)$ over the radial distance.

The bending energy contribution is defined by:

(5) $$\Phi_{angle}\left(\varphi \right)=\frac{1}{2}k_{B}{\left(\varphi -\varphi_{i}\right)}^{2}$$

with $k_{B}$ related to the bending stiffness of a tropocollagen molecule.^23^, ^34^ The parameter $\varphi_{i}$ represents the equilibrium angle between two beads of the coarse‐grained model. Several different equilibrium angles were selected in order to mimic a collagen molecule's initial geometry (Fig. 1 *A*). The angles were measured on the coarse‐grained model of the mineralized collagen molecule and rounded to the nearest integer to reduce the number of variables. This resulted in 13 distinct equilibrium angles ranging from 164 to 180 degrees.

Intermolecular interactions are modeled by a generic Lennard‐Jones (LJ) potential:

(6) $$E_{non-bonded}\left(r\right)=4\epsilon_{coll}\left[{\left(\frac{\sigma_{coll}}{r}\right)}^{12}-{\left(\frac{\sigma_{coll}}{r}\right)}^{6}\right]$$

where $\sigma_{coll}$ is the distance parameter and $\epsilon_{coll}$ the energy parameter which determines the strength of the intermolecular adhesion without the presence of any crosslinks.^23^

Similarly, the energy contribution of hydroxyapatite, $E_{HAP}$, and of the interaction between collagen and mineral particles, $E_{inter}$, are described by a LJ potential with the parameters $\sigma_{HAP}$ and $\epsilon_{HAP}$ for hydroxyapatite and $\sigma_{inter}$ and $\epsilon_{inter}$ for the interaction. Here we focus on the behavior of dry collagen fibrils, to be consistent with the full atomistic simulations we used to parameterize the geometry of our coarse‐grained model.^32^, ^37^ Besides, it has been shown that water reacts with hydroxyapatite to form a hydrated layer around the crystal.^38^ Some preliminary data suggest that the interaction between collagen and mineral can vary significantly depending on the amount of water present in this hydrated layer (data not shown). To minimize these variations, the parameters for collagen‐mineral interaction have been derived in vacuum.

The total potential energy of the system is given by the sum of all pairwise and three‐body interaction:

(7) $$E_{bond}=\underset{bonds}{\sum }\Phi_{\text{bond}}\left(r\right);\;E_{angle}=\underset{angles}{\sum }\Phi_{angle}\left(\varphi \right);\;E_{I}=\underset{pairs}{\sum }\Phi_{I}\left(r\right)$$

with I={coll,HAP,inter}.

Here, we focused on quasi‐static mechanics of a collagen fibril. Therefore, no viscosity has been included in the model. The parameters of the coarse‐grained model are summarized in Table 1.

**Table 1:** Summary of the Parameters Used in the Mesoscopic Molecular Model of Mineralized Collagen Fibrils

Component	Parameter	Value
Collagen	Equilibrium bead distance *r_0_*, Å	14.00
	Critical hyperelastic distance *r_1_*, Å	18.20
	Bond breaking distance *r_break_*, Å	21.00
	Tensile stiffness parameter *k_T_^(0)^*, kcal.mol^–1^.Å^−2^	17.13
	Tensile stiffness parameter *k_T_^(1)^*, kcal.mol^–1^.Å^−2^	97.66
	Equilibrium angle, *θ_0_*, degree	164−180.00
	Bending stiffness parameter *k_B_*, kcal.mol^–1^.rad^–2^	14.98
	Dispersive parameter *ϵ_coll_*, kcal.mol^–1^	6.87
	Dispersive parameter *σ_coll_*, Å	14.72
Hydroxyapatite	Mass of mesoscale particle, atomic mass units	1548
	Dispersive parameter *ϵ_HAP_*, kcal.mol^–1^	106.7
	Dispersive parameter *σ_HAP_*, Å	10.28
	Cutoff radius, *c_HAP_*, Å	13.85
	Mass of mesoscale particle, atomic mass units	1324
Interaction	Dispersive parameter *ϵ_inter_*, kcal.mol^–1^	137.1
	Dispersive parameter *σ_inter_*, Å	9.88
	Cutoff radius, *c_inter_*, Å	20.00

1 kcal.mol^–1^.Å^−1^ = 69.479pN.

### Modeling procedure

All molecular dynamics simulations have been performed using the Large‐scale Atomic/Molecular Massively Parallel Simulator (LAMMPS) code.^39^ For the mineralized fibril simulation, the model is equilibrated in vacuum for 20 ns. An isothermal‐isobaric (NPT) ensemble (which keeps the number of particles [N], system pressure [P], and temperature [T] constant/conserved) was used to allow the relaxation of the fibril along its length. After complete relaxation, confirmed by a steady root mean‐square deviation, the collagen fibril was stretched by deforming the simulation box with a displacement rate of 1 m/s. Such high strain rates, compared to rates used in experimental testing, are a consequence of the timescale limitation of the molecular model; total time‐spans of several microseconds are the most that can be simulated because the time‐step has to be on the order of several femtoseconds. The integration time step is set to Δt = 10 fs and the temperature to 300°K. We use the virial stress to compute the stress tensor^40^ for analyses of the strain‐stress behavior of the fibril and collagen molecules.

### Visualization and data analysis

**Figure 2:**
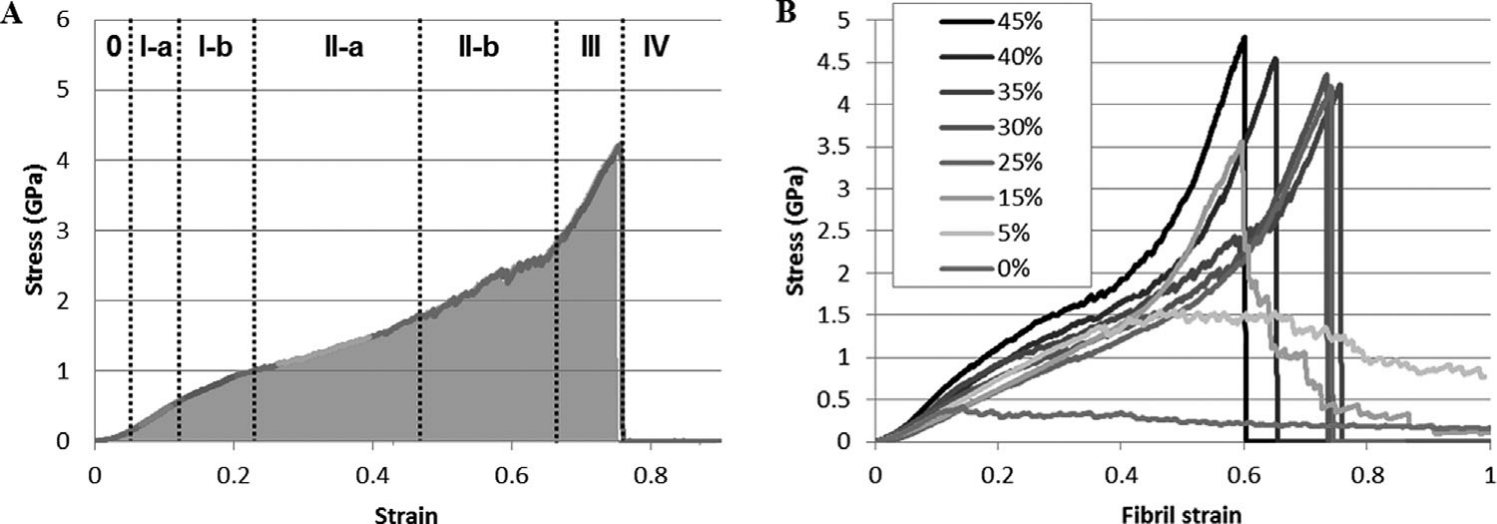
(*A*) Representative stress‐strain response of a mineralized collagen fibril containing 35% mineral. Regions 0 to IV represent the different deformation regimes exhibited by the fibril. The gray area has been used to compute the fibril's toughness. (*B*) Strain‐strain curves until rupture of collagen fibril containing different mineral densities.

The coordinates of the particles in the coarse‐grained models were created with Matlab R2010a (Mathworks Inc., Natick, MA, USA) based on the full atomistic geometry described previously.^32^ Visualization of the simulation is performed with visual molecular dynamics (VMD).^41^ Stress‐strain curves were computed from the box size and the virial‐stress of the particles. The diameter of the fibril was considered constant and equal to 20 nm. The stress‐strain response was divided into five regions to analyze the mechanical response of the fibril (Fig. 2 *A*). The toughness of the fibril is defined as the area under the stress‐strain curve until ultimate strength (gray area in Fig. 2 *A*).

All mineral densities are expressed as weight percentages of the total model.

## Results and Discussion

In this study, we explored the large‐scale deformation and fracture behavior of a single mineralized collagen fibril under tensile loading. The model used here is based on previous full‐atomistic simulations and assumes that most of the mineral within the fibril lies in the gap regions of the fibril. The deformation regimes are induced by several mechanisms including elastic deformation, mineral‐collagen sliding, intermolecular slippage, and mineral dissociation. In the following sections, we discuss the mechanisms highlighted in this study.

### Mechanisms of elastic and inelastic deformation of mineralized collagen fibrils

**Figure 3:**
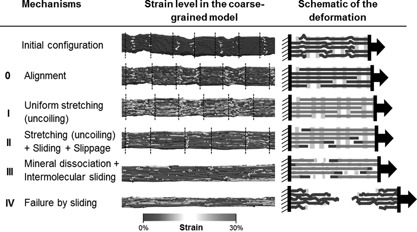
Representative deformation mechanisms and molecular strain level for a low mineralized collagen fibril (5%). The initial configuration of the fibril presents a wavy shape due to crimping. Two distinct mineralized clusters are visible in each gap region (delimited by dashed lines). (0) The first 5% of the deformation corresponds to the uncrimping of the fibril along the pulling direction, which corresponds to the toe region of the strain‐stress curve. (I) The fibril enters an elastic regime where the collagen molecules display a slightly higher strain level in the gap than in the overlap region because of the lower molecular density. (II) At around 15% of deformation, some molecular slippage can be observed resulting in the relaxation of molecular termini. The remaining molecules continue to sustain elastic deformation and are combined with collagen‐mineral and mineral‐mineral sliding. (III) Further increasing the deformation of the fibril leads to the dissociation of mineral clusters, which favors molecular sliding. (IV) Molecular sliding ultimately leads to the fibril rupture.

**Figure 4:**
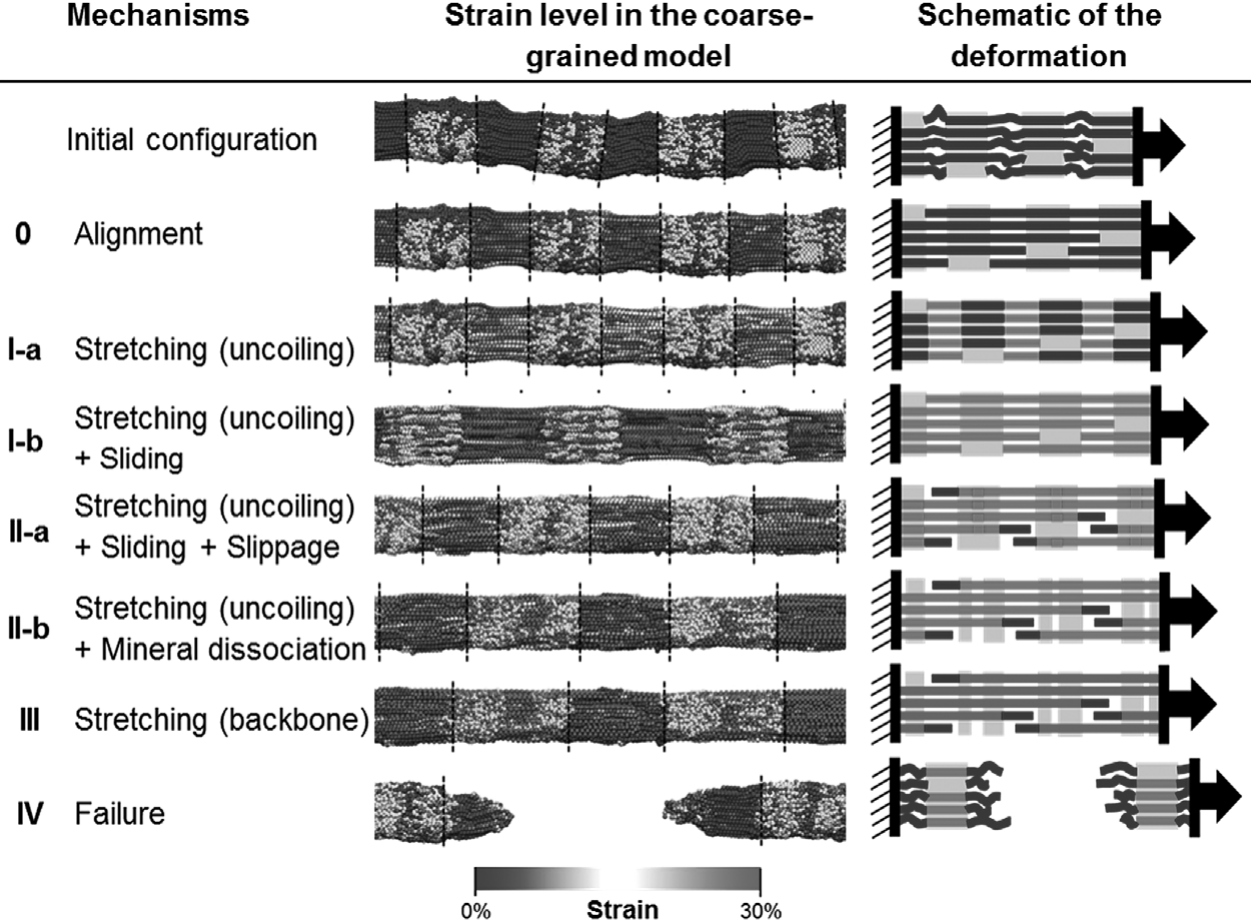
Deformation mechanisms for high‐density mineralized collagen fibrils (35%) showing all possible deformation mechanisms that can arise during tensile loading. (0) The first 5% of the deformation corresponds to the uncrimping of the fibril. (I‐a) The fibril enters an elastic regime. Due to the presence of mineral, most of the deformation arises in the overlap region. (I‐b) Due to some sliding between mineral and collagen, the load is transferred the collagen molecules. (II‐a) At around 20% strain, molecular slippage occurs resulting in the relaxation of molecular termini, proving that collagen‐mineral adhesion energy is not enough to sustain the load. The remaining molecules continue to sustain elastic deformation and collagen‐mineral and mineral‐mineral sliding occurs in the gap regions. (II‐b) Mineral phase dissociates into two clusters per gap region. Molecular strain in the gap region increases to reach to levels observed in the overlap region. (III) Collagen molecules enter their second deformation regime corresponding to their backbone stretching. (IV) The molecules reach their failure strain and break leading to the catastrophic failure of the fibril.

The fibrils were tested by tensile loading until 100% strain. A representative stress‐strain curve of a mineralized fibril in tension is presented in Fig. 2 *A*, whereas Fig. 2 *B* summarizes the stress‐strain behavior of collagen fibrils containing different mineral densities. During tensile loading, a mineralized collagen fibril can undergo five to seven different regimes depending on the mineral density in the fibril. These regimes reflect the different energy dissipation mechanisms in play during fibril stretching. It includes elastic stretching, mineral/collagen sliding, molecular slippage, and mineral dissociation. Figures 3 and 4 represent the characteristic behavior of mineralized fibrils containing a low‐density and high‐density mineral. The mechanisms behind the different regimes are presented in the following sections.

#### Mineral‐induced fibril contraction

**Figure 5:**
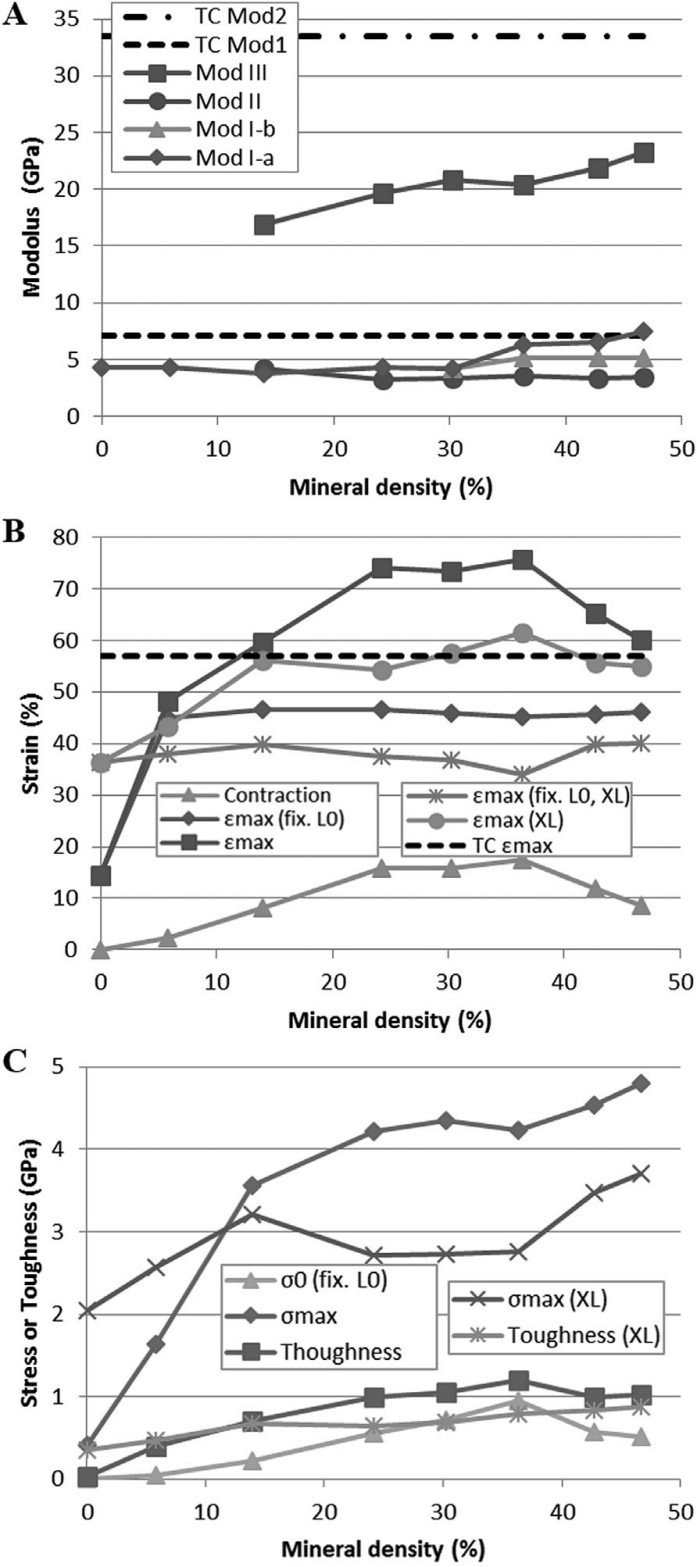
Evolution of the mechanical properties of a collagen fibril depending on their mineral density. (*A*) Characteristic moduli of the mineralized fibrils for regime I, II, and III (Mod I‐a, I‐b, II, and III). The dashed lines represent the characteristic moduli for a single tropocollagen molecule (TC Mod1 and Mod2). (*B*) Amount of fibril contraction due to the introduction of mineral. The maximum contraction appears for mineral densities between 25% and 35% and reaches almost 20%. Ultimate strain for variable initial length $\epsilon_{max}$, and assuming an identical initial length $L_{0}$ for all samples $\epsilon_{max}$ (fix. L_0_) where $L_{0}$ is the initial length of a nonmineralized collagen fibril. Both ultimate strains are also reported for cross‐linked fibrils ($\epsilon_{max}$ (XL) and $\varepsilon_{max}$ (fix. L_0_, XL)). The dashed line represents the ultimate strain of a single tropocollagen molecules (TC $\epsilon_{max}$). (*C*) Ultimate stress $\sigma_{max}$, toughness and fibril internal stress at the length $L=L_{0}$, $\sigma_{0}$ (fix. L_0_). Fibril crimping induces internal stresses in the fibrils up to 1 GPa. The values of ultimate stress and toughness are also presented for cross‐linked fibrils ($\sigma_{max}$ (XL) and toughness (XL)).

Introducing mineral in the fibril leads to a decrease in the sample's initial length. This contraction is dependent on the mineral density. Compared to the length of pure collagen fibril, mineralized collagen fibril length can decrease up to 17% (Fig. 5 *B*). This is in good agreement with experimental observations which show mineralization results in an axial contraction of collagen fibril, and also show that this contraction occurs mainly in the gap region of the fibril where mineralization occurs.^42^ In the model, the mineralization of the fibril starts in the gap region near each terminal domain of the molecules. For low mineral densities, this results in two distinct crystals per gap region (Figs. 1 and 3). Due to large interactions, these crystals are attracted to each other. Because the crystals are strongly connected to collagen, this leads to a decrease in the size of the gap region and therefore of the fibril. As mineral density increases, the size of the two crystals increases, leading to larger interactions and therefore larger crimping of the fibril. The fibril contraction reaches its maximum for a mineral density of 35%, where the two crystals merge after equilibration. For higher mineral densities, the crystals are larger and the mineralized gap region become stiffer, leading to lower contraction values. Above 35% mineral densities, the two initial crystals merge after equilibration, forming one single crystal in each gap region (Figs. 1 and 4). Assuming the fibril keeps its initial length during the mineralization process, adding mineral induces internal stresses. The internal stress can reach 1 GPa for 35% mineral and follows the same trend as fibril contraction (Fig. 5 *C*). The first 5% strain of the fibril corresponds to a toe region due to uncrimping of the fibril (Figs. 2, 3 and 4, regime 0).

#### Improvement of collagen fibril's elastic behavior by mineralization

After the toe region, a mineralized collagen fibril exhibits a linear deformation regime (Fig. 2 *A*, regime I‐a). The stiffness of the elastic regime is dependent on the mineral's density. Below 15% mineral, the presence of mineral does not significantly influence a fibril's stiffness compared to pure collagen fibril because they display a similar Young modulus (Mod I‐a, E=3.71±0.35 GPa) (Fig. 5 *A*). Above 15% mineral, the elastic modulus increases as a function of mineral density and plateaus around 30% (Mod I‐a, E=7.96±0.13 GPa), which is similar to the modulus of a single tropocollagen molecule (E≈7 GPa).^23^

**Figure 6:**
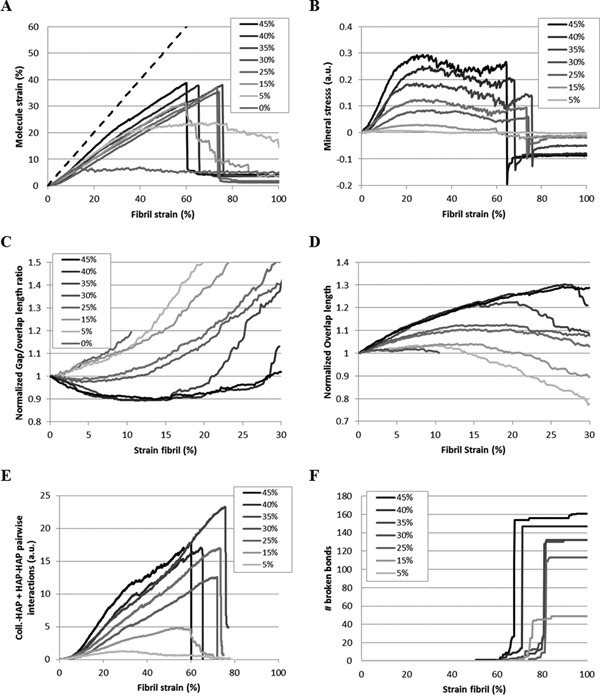
(*A*) Average strain and (*B*) mineral internal stress at the molecular level in fibrils containing various densities of mineral as a function of fibril strain. (*C*) Normalized gap/overlap length ratio and (*D*) normalized overlap length in the fibrils. Gap and overlap length are measured based on the number of molecules in the fibril cross‐section. (*E*) Sum of pairwise interactions between collagen‐mineral beads and mineral‐mineral beads as a function of fibril strain. The decrease of the slope arising at the beginning of regime II is consistent with sliding between the different components of the fibrils. (*F*) Evolution of the number of broken collagen bonds in the fibril as a function of applied strain for varying mineral density.

In the model, mineral crystals are formed near the N‐ and C‐terminal domains of the fibril. Until 30% mineral, two distinct crystals are present in the gap region, the middle of the gap region remaining unmineralized. Because the gap region contains fewer molecules than the overlap region, deformation is predominant in the gap region, which explains the low modulus below 10% mineral. Above 30% mineral, the two crystals in the gap region merge. In this case, the load in the gap region is transferred to the continuous mineral phase. Because the mineralized gap region is stiffer than the overlap region, which contains only collagen, fibril deformation happens mostly in the overlap region (Fig. 6 *C*, *D*). Because the overlap region contains more collagen molecules along its cross‐section compare to the gap region, and because the stress is computed based on a constant cross‐section of the fibril, the fibril's initial apparent stiffness is increased (Fig. 5 *A*, Mod I‐a).

During regime II‐a, the mineral's internal stress increases linearly (Fig. 6 *B*). In the case of a pure collagen fibril, the collagen molecules reach a strain of 10% before the next deformation regime sets in. Due to the presence of mineral, intermolecular adhesion is increased, and when compared to a pure collagen fibril, mineral allows an increase of fibril yield by delaying intermolecular sliding. For a mineralized fibril, yield occurs between 10% and 20% strain. Even for low mineral densities, where the phenomenon is limited, both yield stress and yield strain are greatly increased. Because mineral is present at the N‐ and C‐terminal domains, its presence leads to an increase in the connectivity of the molecules similar to cross‐linkage.^43^ Yielding occurs when the adhesion energy at the collagen–mineral interface become insufficient to sustain tensile loading. At this point, the link between the terminal region of the collagen molecules and mineral is disrupted and intermolecular sliding/slippage takes place.

#### Load transfer from mineral to collagen

For high mineral density (>30%), a second elastic regime starts between 12% and 15% (Fig. 2 *A* and 4, regime I‐b). This sub‐regime is characterized by a lower elastic modulus compared to the first part of the regime (II‐a) (Mod I‐b, E=5.15±0.02GPa) (Fig. 5 *A*). During this regime, the gap/overlap ratio increases, indicating that the deformation become predominant in the gap region (Fig. 6 *C*). The slope of the mineral stress decreases, showing a reduced load transfer between the collagen molecules and the mineral crystals (Fig. 6 *B*). The modulus of this regime is similar to the one of regime I‐a of low‐density fibrils, indicating that load in the gap region is transferred back to the collagen.

#### Molecular slippage and mineral‐collagen sliding

A mineralized fibril exhibits a three‐phase behavior similar to what was observed for a crosslinked fibril.^43^, ^44^ Between ∼25% and 40% strain, the stress‐strain curve exhibits a linear deformation regime displaying a lower modulus (Fig. 5 *A*, Mod II, E=3.54±0.35GPa) and a saw‐tooth shape, which is characteristic of repeated intermolecular slippage and sliding (Fig. 2). The adhesion energy between collagen molecules and mineral crystal is not sufficient to sustain the tensile load, as proven by collagen termini slippage. This phenomenon is associated with collagen‐mineral and mineral‐mineral sliding, as proven by a decrease in the slope of the pairwise interaction between collagen and mineral (Fig. 6 *E*). The load energy accumulated in the mineral phase is transferred to the collagen matrix by a series of sliding events. This results in a saw‐toothed decrease of mineral longitudinal stress (Fig. 6 *B*).

The length of the third deformation regime is proportional to the crimping of the fibril because part of this contraction occurs in mineralized areas due to strong mineral‐mineral interactions.

**Figure 7:**
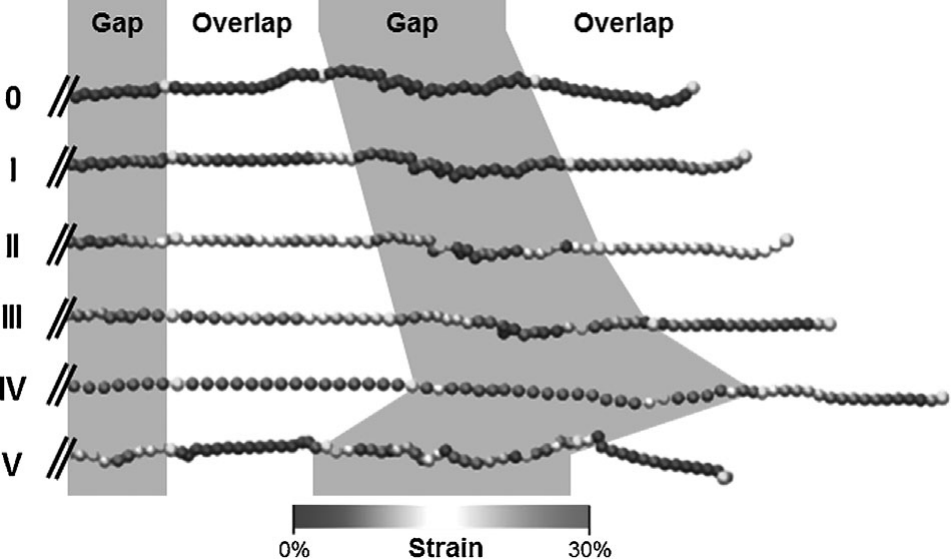
Strain in the terminal domain of a collagen molecule taken from a mineralized fibril containing 35% mineral for each deformation regime (I to V) of the tensile test. The yellow beads mark the transitions between gap and overlap regions. In regime II, the terminal domain of the molecules is relaxed due to molecular slippage. The other overlap region remains loaded due to the clamping of its boundaries by mineral crystals (not shown).

Molecular slippage can be observed only in the overlap regions of the fibril, which contain terminal regions of the tropocollagen molecules (Figs. 3 and 4, regime II). Indeed, collagen termini, as free ends of the molecule, allow for a relaxation of the triple helix (Fig. 7, II). On the other side of the overlap region, the triple helix is clamped in mineral and the rest of the molecule remains stressed. This also leads to a decrease of the average molecular strain (Fig. 6 *A*). Because the gap and overlap length are measured based on the number of molecules across the fibril's cross‐section, the decrease in overlap length is consistent with molecular slipping (Fig. 6 *D*).

#### Mineral crystal dissociation as an energy dissipation mechanism

In highly mineralized fibrils (density >30%), the mineral phase in the gap region forms a single crystal after equilibration (Figs. 1 and 4). During previous deformation regimes, the load applied to the fibril is transferred from the collagen of the overlap region to the mineral phase present in the gap region. Between 35% and 45% strain, depending on the mineral density, apatite crystals present some signs of dissociation, as can be inferred from the stress‐strain curve and mineral's internal stress’ large saw‐tooth shape (Figs. 2 *B* and 6 *B*). Crystal dissociation can also been observed in Fig. 4, where regime II presents one single mineral phase per gap region whereas regime II and III present two separate crystal clusters. During that regime, mineral loading is transferred back to collagen as seen in Fig. 7. Indeed, the beads in the gap region that are unloaded during regime II become highly strained in regime III, which confirms the load transfer.

#### Collagen fibril stiffening enhanced by large mineral densities

When most of the possible intermolecular slip has been exhausted, fibrils with a mineral density larger than 10% reach a fourth deformation regime (Fig. 2, regime III). No more intermolecular slippage is possible and collagen molecules and mineral crystals sustain the tensile load cooperatively. Both molecular stain and mineral stress increase (Fig. 6 *A*, *B*). If we assume fibril initial length to be the same at the beginning of the simulation, all the fibrils enter the fourth deformation regime (III) at around 30% deformation, which corresponds to the initial strain of the second deformation mechanism of a single collagen molecule (Supporting Information, Fig. 4 *B*). The fourth regime is characterized by greater stiffness, linearly dependent on mineral content, and ranging from 16.3 to 23.2 GPa (Mod III, Fig. 5 *A*). The stiffness of the fourth regime tends toward the single molecule's second regime's stiffness of 33.5 GPa. Regime III corresponds to the initiation of triple helix backbone stretching and proves that collagen molecules form a network that deforms synergistically. The modulus of a single tropocollagen is never reached because some of the molecules are in a relaxed state after the slippage mechanism and do not contribute to the load resistance.

### Improved fibril strength, extensibility and toughness due to mineral crystals

The behavior of a fibril varies depending on its mineral density. For low mineral densities (≤5%), a fibril presents a ductile comportment similar to pure collagen fibril. Intermolecular sliding occurs as mineral crystals lose their integrity (Fig. 3, regime III). Similar to a pure collagen fibril, mineralized fibrils with low mineral density fail through intermolecular sliding. However, the ultimate stress $\sigma_{max}$, ultimate strain $\epsilon_{max}$ and toughness are largely increased. For a mineral density of 5%, $\sigma_{max}$ and $\epsilon_{max}$ are tripled and the toughness is multiplied by a factor of 10 when compared to pure collagen fibril values. In the process, only a few collagen molecules reach their failure point (Fig. 6 *F*).

For higher mineral densities, a fibril reaches its maximum stress at the end of regime III and exhibits a brittle‐like behavior with very little plastic deformation. The ductile/brittle transition arises for a mineral density around 15%. For that density, the crystals lose their cohesion during regime III and the failure of the fibril is a mix between intermolecular sliding (Fig. 6 *A*) and tropocollagen molecules failure (Fig. 6 *F*). Around a 25% mineral density, a fibril reaches its optimal properties and adding mineral does not seem to significantly improve the fibril's tensile behavior. The ultimate stress and toughness reach a plateau with a mean value of 4.43 ± 0.25 GPa and 1.05 ± 0.08 GPa, respectively (Fig. 5 *B*, *C*).

When compared to pure collagen fibril, the mineral phase leads to a drastic increase in the fibril's mechanical properties. The values of $\epsilon_{max}$, $\sigma_{max}$, and the toughness are on average multiplied by factors of 3, 10, and 35, respectively. Assuming a fixed initial length L_0_, all fibrils with a density above 25% fail at a strain of 45.9 ± 0.5% which is around 20% lower than a single tropocollagen molecule's ultimate strain (Fig. 5 *B*). If the fibril is allowed to relax at the beginning of the simulation, the ultimate strain can reach 75% for mineral densities between 25% and 35%. At failure, at least four‐fifths of the molecules break, which corresponds to the number of collagen molecules in the cross‐section of the fibril overlap region that are not subject to slippage (Fig. 6 *F*). The more mineral in the fibril, the more molecules break due to increasingly constrained tropocollagen molecules. After rupture, the fibril presents some residual stresses in both organic and mineral phases (Fig. 6 *A*, *B*, and Fig. 7). Residual stresses are proportional to the mineral density and attest to some mineral sliding during the loading of the fibril.

### Crosslinks and mineralization

**Figure 8:**
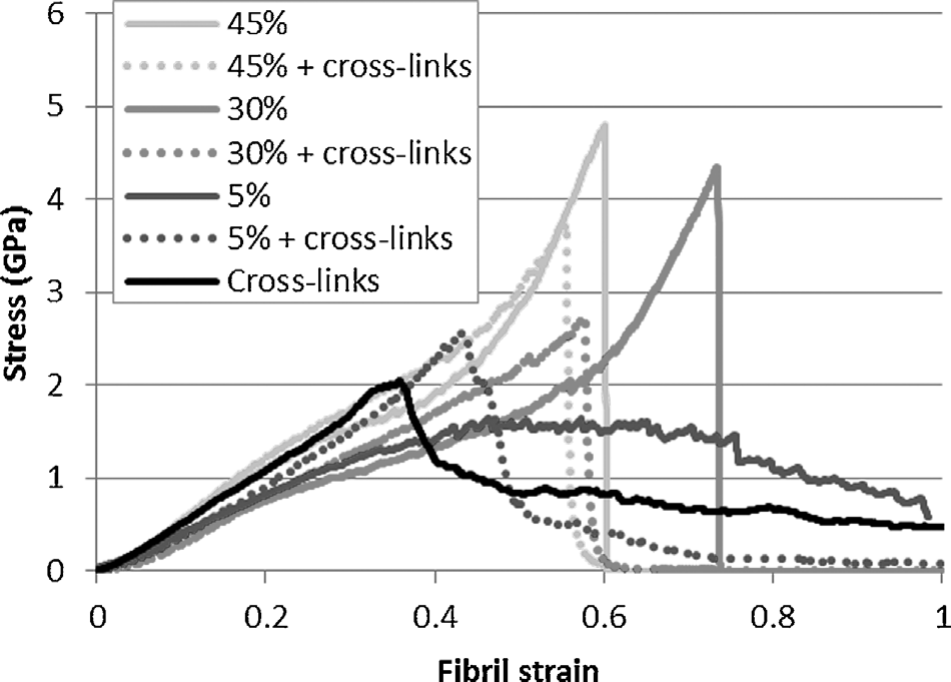
Stress–strain response of a collagen fibril containing different density of intrafibrillar mineral (plain line). The same models are presented with two trivalent cross‐links per molecule (dashed line). Combining cross‐links with intrafibrillar mineralization improves the second deformation regime by limiting molecular slippage but decreases the failure strain by overconstraining the structure.

Adding crosslinks in a mineralized collagen fibril significantly alters its mechanical response (Fig. 8). For low mineral densities (∼5%), crosslinking mainly dictates the mechanical response of the fibril. The stiffnesses of deformation regimes I, II, and III are not significantly different compared to fibrils containing only crosslinks. However, the combination of crosslinks and mineral delays the failure, which yields an improvement of both strength and elasticity in comparison to purely crosslinked fibrils (Fig. 5 *C*). For larger mineral densities (above 15%), the addition of crosslinks improves the cohesion between the molecules, which prevents the slippage of terminal domain of tropocollagen molecules when compared to a fibril with mineral alone (Fig. 8). This results in an increase of regime II elastic modulus, which reaches the value of regime I‐a of low‐density fibrils (4.6 ± 0.3 GPa versus 4.2 ± 0.2 GPa). This indicates that the load is sustained mainly by the collagen structure and that most of the molecules are deformed cooperatively due to the presence of the crosslinks. Failure arises at significantly lower strain as the presence of crosslinks in the fibril create an overconstrained structure limiting the mobility of the molecules (Fig. 5 *B*). By taking into consideration the crimping of the fibril induced by the mineral for all samples, the failure strain matches the one of an unmineralized crosslinked fibril (37.9% ± 2.0% versus 36.4%, Fig. 5 *B*). This attests that the crosslinks drive the failure of the crosslinked mineralized fibrils. As a consequence, a crosslinked mineralized fibril with a density above 15% exhibits significantly lower failure strain, strength, and toughness compared to a purely mineralized fibril (Fig. 5 *B*, *C*).

### Strengthening, toughening, and damage confinement

The introduction of mineral in a collagen fibril greatly improves the fibril's mechanical properties without disturbing the integrity of the fibrillar structure, which still displays its characteristic banding pattern. Our results suggest that a mineralized fibril presents up to five energy dissipation regimes, which lead to a drastic increase in the strength and total amount of energy dissipated before failure. By adding intrafibrillar mineral, the strength of a fibril can be increased up to 10 times when compared to a nonmineralized fibril. The energy dissipated during tensile loading can be 35 times greater than pure collagen.

Adding a large number of crosslinks to a mineralized collagen fibril improves the cohesion of the molecular structure. It results in an improved stiffness in the large deformation regimes but limits the mobility of the molecules, which leads to lower ultimate strain and strength of the fibril, confirming that a large mature crosslink density could be deleterious for bone strength.^45^

**Figure 9:**
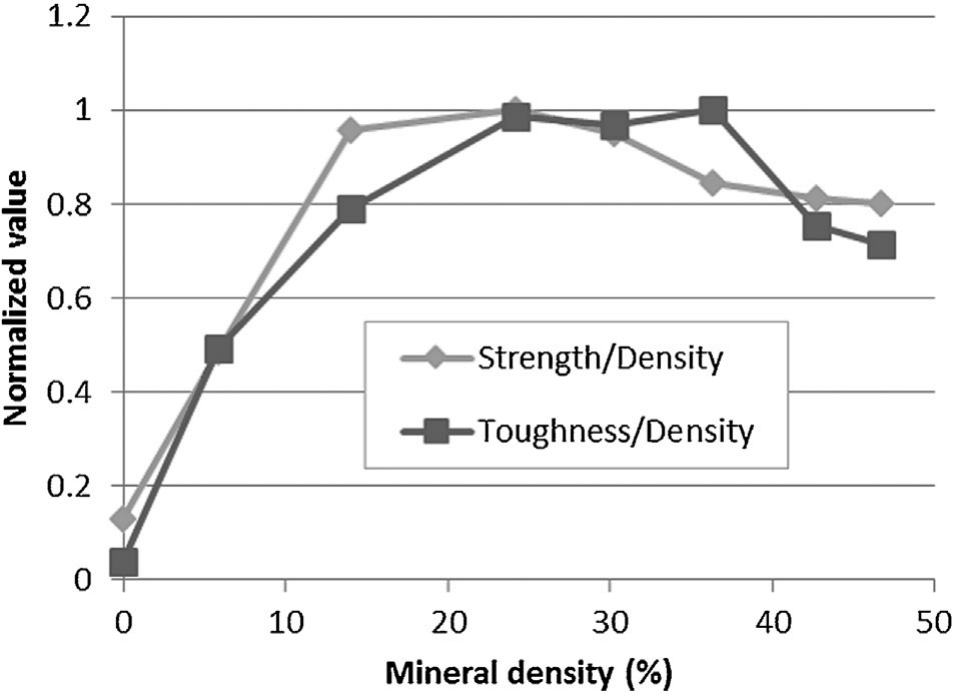
Fibril toughness and strength normalized by the fibril density and as a function of mineral density. Both toughness and strength are optimized for fibril density for a mineral density around 30%.

In this study, we found that the optimal amount of mineral maximizing both toughness and strength of a single mineralized collagen fibril while maintaining a low density lies around 30% (Fig. 9). This observation is in good agreement with densities measured experimentally^46^, ^47^, ^48^ and supports the idea that bone tissue nanostructure has evolved in order to be strong yet lightweight to facilitate movement and mobility.^3^

Deformation mechanisms such as sliding and mineral dissociation allow large energy dissipation along the whole length of the fibril without significantly disturbing the fibrillar structure. These mechanisms allow the delocalization of the stress along the whole fibril, avoiding stress concentrations and favoring large energy dissipation. Ultimately, most of the fibrils undergo brittle‐like failure, keeping the rest of the fibril minimally altered. This failure mechanism appears to be a biological response to delocalize damage and maintain structural integrity during large deformation. Keeping the fibrillar organization could also favor pullout mechanisms, which represent yet another energy dissipation mechanism at the tissue scale, as observed in mineralized tendon^49^ or at bone fracture surfaces.^14^, ^50^, ^51^

## Conclusion and Perspectives

In this study, we present a model for the deformation mechanisms of mineralized collagen fibril subject to tensile loading. Our model sheds light on the nanoscale deformation mechanisms taking place in the structure of a mineralized fibril. Under tension, a fibril can undergo up to five sequential deformation mechanisms as follows:

1. An initial elastic deformation corresponding to the collagen molecule uncoiling;
2. A second regime where mineral/collagen sliding is predominant;
3. A linear regime characterized by molecular slippage;
4. A regime where mineral dissociation leads to a load transfer back to the collagen; and
5. A final linear regime linked to the stretching of the backbone of collagen molecules until fibril failure.

This succession of dissipation mechanisms leads to a drastic increase in fibril's mechanical properties; the strength and toughness are increased by a factor of 10 and 35, respectively, when compared to a nonmineralized fibril.

We found that the mineral density maximizing a fibril's strength and toughness while minimizing fibril density is around 30%. Similar values have been observed experimentally supporting the idea that nature tends to optimize structure of bone at the nanoscale to form a strong, tough, and lightweight material.

Adding crosslinks to a mineralized fibril affects its mechanical response. For low mineral densities, the fibril response is similar to the response of a fibril with crosslinks alone with improved strain and stress to failure. For large densities, adding crosslinks increases the stiffness of the second deformation regime (regime II) by preventing the slippage of the tropocollagen molecules. On the other hand, the crosslinks reduce the failure strain by overconstraining the fibrillar structure, making the fibril more brittle.

The model presented here opens some new possibilities to explore the relationship between collagen fibril composition and nanostructure, and the fibril nanomechanical response. Understanding the basic building block of bone, the mineralized fibril, is crucial in determining the unique mechanical properties of bone tissue, defining mechanism of diseases that affect the mechanical integrity of bone, and designing biomimetic structures and biomaterials that capture the salient features of the mineralized fibril.

## Disclosures

All authors state that they have no conflicts of interest.

## Supporting information

Supporting Information.
