# Supplementary material for: Large Deformation Mechanisms, Plasticity, and Failure of an Individual Collagen Fibril With Different Mineral Content
Source: J Bone Miner Res. 2016 Feb 11;31(2):380–90. doi: 10.1002/jbmr.2705 (PMC4915725; doi:10.1002/jbmr.2705)
Supplement: Supplementary file 1 — Supporting Information. [file JBMR-31-380-s001.pdf]

Large deformation mechanisms, plasticity and failure of finite size mineralized collagen fibrils – Supporting Information

Baptiste Depalle[1], Zhao Qin[1], Sandra J. Shefelbine[2], Markus J. Buehler[1,3,4]*

[1] Laboratory for Atomistic and Molecular Mechanics (LAMM), Department of Civil and Environmental Engineering, Massachusetts Institute of Technology, 77 Massachusetts Ave., Room235 A&B, Cambridge, 02139 MA, USA
[2] Department of Mechanical and Industrial Engineering, Northeastern University, Boston, MA USA
[3] Center for Computational Engineering, Massachusetts Institute of Technology, 77 Massachusetts, Ave. Cambridge, MA 02139, USA
[4] Center for Materials Science and Engineering, Massachusetts Institute of Technology, 77 Massachusetts Ave., Cambridge, MA 02139, USA

* Corresponding author: mbuehler@mit.edu

1 Material and Methods

1.1 Derivation of parameters for mineral interactions

The interfibrillar mineral is modeled by an FCC lattice. Since hydroxyapatite is an ionic crystal, the complex interactions between the ions are approximated by a Lennard-Jones potential Eq. (6). The equilibrium distance between two particles is given by:

$$r_0 = 2^{1/6}\sigma_{HAP} \tag{8}$$

For an FCC lattice, it corresponds to the nearest neighbor's distance which is:

$$r_0 = 2^{-1/2}a_0 \tag{9}$$

Thus:

$$\sigma_{HAP} = 2^{-2/3}a_0 \tag{10}$$

The stiffness of the LJ potential is given by:

$$k = \phi''(r_{ij} = r_0) = \frac{d^2\phi}{dr_{ij}^2}|r_{ij} = r_0 = \frac{4\varepsilon_{\mathrm{HAP}}}{r_0^2}\left[156\left(\frac{\sigma_{\mathrm{HAP}}}{r_0}\right)^{12} - 42\left(\frac{\sigma_{\mathrm{HAP}}}{r_0}\right)^{6}\right] \tag{11}$$

Since $r_0 = 2^{1/6}\sigma_{HAP}$:

$$k = \frac{72\varepsilon}{2^{1/3}\sigma_{HAP}^2} \tag{12}$$

The bulk modulus is given by:

$$K = \frac{E}{3(1-2\nu)} \tag{13}$$

With E the elastic modulus and ν, the Poisson ratio.
Assuming that elasticity is due to energetic contributions and that hydroxyapatite satisfies the Cauchy-Born rules, the shear modulus of the FCC crystal can be expressed as [1]:

$$\mu = \frac{r_0^2 k}{2V} \tag{14}$$

With V the volume of a unit cell which, for a FCC lattice equals to $V = a_0^3/4$ since there are 4 atoms per unit cell.
Combining Eq. (11), (13) and (14) and using the relationship $E = \frac{8}{3}\mu$, we obtain:

$$\varepsilon_{HAP} = \frac{(1-2\nu)Ka_0^3}{128} \tag{15}$$

The cutoff radius of the LJ potential is chosen to be 20% larger than the nearest neighbor distance.
Interactions between collagen and apatite are also represented by a LJ potential. The equilibrium distance is assumed to be equal to a single tropocollagen molecule's radius. At equilibrium, a collagen particle will be located at the apex of a square pyramid with a base formed by 4 hydroxyapatite atoms (**Fig. S1A**). The base diagonal is equal to the FCC lattice

(a_0) and its height corresponds to the desired equilibrium distance r_{inter}. The equilibrium distance b_0 between collagen and mineral particle is determined by geometric relations and σ_{inter} is found according to Eq. (10).
The energy parameter ε_{inter} and the cutoff radius c_{inter} are computed by direct comparison between full atomistic and coarse-grained simulation of collagen-hydroxyapatite adhesion. Steered molecular dynamic (SMD) have been used to explore the shear strength of collagen-mineral composite (**Figs. S1B and C**). To overcome the loading rate dependence, the shear strength has been computed for several loading rates ranging from 1 to 20 m/s and extrapolated to a quasi-static loading (0 m/s) (**Fig. S1D**). Using an iterative scheme, the coarse-grained parameters ε_{inter} and c_{inter} are selected to match the quasi-static shear strength.

1.2 Fibril model geometry

The geometry of the coarse-grained models is based on full atomistic simulation of mineralized collagen previously reported [(2)]. Further details on the development of the mineralized collagen fibril full atomistic model can be found in references [(2,3)]. Briefly, the tropocollagen geometry is based on Protein Data Bank entry 3HR2 representing the microfibrillar structure of type I collagen measured in situ by X-ray crystallography [(4)]. The fibril is mineralized *in silico* by filling the gaps in the model with hydroxyapatite ions. The model takes only intrafibrillar mineralization into account. According to the literature, extrafibrillar mineral fraction can represent most of the total mineral in bone tissue [(5,6)]. We create fibril models including up to 45% in weight of intrafibrillar mineral, which represents the upper limit of the density naturally found in bone.
The conversion from full atomistic to coarse-grained is performed in two steps. For the protein matrix, the coordinates of tropocollagen molecule's atoms are averaged using spline approximation along the length of the molecule. Equidistant beads are then created along the spline to create the coarse-grained model of the collagen molecules with an equilibrium distance r_0 = 14.0 Å (**Fig. 1**). In order to vary the mineral density inside the fibril, several full atomistic models of crystals are created based on a distance criterion. By removing the hydroxyapatite atoms which are within a distance cutoff from any tropocollagen molecules, we are able to adjust the crystal size, and consequently reach various degrees of mineralization. Full atomistic model of crystals corresponding to densities ranging from 5 to 45% have been created. To convert the full-atomistic crystals into a coarse-grained representation, a face-centered cubic (FCC) lattice is created with a lattice distance a_0 = 16.322 Å which corresponds to the thickness of two hydroxyapatite unit cell (**Fig. 1**). This way, two layers of the FCC lattice are separated by the equivalent of one hydroxyapatite unit cell thickness. This allows us to conserve the crystal's characteristic thickness which is likely to play an important role in the mechanics of the mineralized collagen fibril. The beads of the FCC lattice are kept if at least 65 atoms of the hydroxyapatite crystal are within a distance $r = a_0 * \sqrt{3}/4$ which is the minimum distance between two beads of the lattice. The fibril is built by replication of the tropocollagen molecule and mineral crystal according to the periodicity given by the full atomistic model. The collagen molecules are replicated in order to form a cylinder of diameter d = 20 nm to represent a fibril. Only the mineral beads within the fibril are kept (**Fig. 2**).
The model of the fibril is built to exhibit five gap/overlap regions along its length which ensures the periodicity of the model along the axis of the fibril (x-axis). During the simulation, the model is replicated by using periodic boundary conditions along its length to simulate an infinitely long fibril. The length of the model is larger than a single molecule length (~300 nm) in order to prevent any artefactual interactions between the two ends of a molecule through the periodic boundaries. The resulting model is made up of 158 molecules which represent a total of 28,305 beads. The number of mineral beads varies between 2,037 and

28,962 depending on the mineral's density. We find that the collagen fibrils naturally display the characteristic staggered arrangement as observed in experiments (**Fig. 2**).

1.3 Modeling procedure

All molecular dynamics simulations have been performed using the LAMMPS code [7].

1.3.1 Full atomistic model

The tropocollagen molecule and crystal structure are generated following the protocol described in [8]. We focused on the interaction of the collagen molecule with the (010) surface which is dominant in bone apatite platelets, due to the growth-directing effect of the collagen matrix [9]. The surface structure has been assumed stoichiometric as described in [10] since it has been observed experimentally in several studies [11,12]. Lennard-Jones and Coulomb interactions are computed with a switching function that ramps the energy and force smoothly to zero starting with 8 Å and cutting off at 10 Å. To model the interaction between collagen and apatite, we used the extended CHARMM force field as presented in [2,8]. We use SMD for pulling the collagen molecule in an *NVT* ensemble (300°K) and an integration timestep of 0.5 fs. We constrained the bottom part of the hydroxyapatite crystal while pulling the center of mass of the α-carbons of each chain of the collagen molecule's right-end (**Fig. S1B**). The simulation is performed in vacuum to be consistent with the full atomistic simulations our model is based on [2] and to limit viscous effects which is necessary with the large strain rate used here.

1.3.2 Mesoscale model

To compute the interaction parameters between collagen and mineral, we recreate the full atomistic model in the coarse-grained representation (**Fig. S1C**). A similar SMD simulation is performed where the last bead of the collagen molecule's right-end is strained with a pulling speed of 1 m/s, similar to that used in previous studies [8,13,14] (**Fig. S1C**).
For all coarse-grained simulations, the integration time step is set to Δt = 10 fs. We use the virial stress to compute the stress tensor [15] for analysis of the strain-stress behavior of the fibril and collagen molecules. All simulations are performed at a temperature of 300°K in an *NVT* ensemble, unless stipulated otherwise.

1.4 Coarse-grained model parameters and validation

To retain the characteristic thickness of mineral crystals, we chose the FCC lattice parameter $a_0 = 16.32$ Å. The bulk properties of hydroxyapatite have been selected according to the literature and set to $\nu = 0.28$ and $K = 90$ GPa. We found $\sigma_{HAP} = 10.28$ Å, $\varepsilon_{HAP} = 193.7$ kcal.mol^{-1} and a cutoff radius $c_{HAP} = 13.85$ Å.
To determine parameters of the Lennard-Jones potential representing the interaction between tropocollagen molecules and hydroxyapatite crystals, the radius of a single collagen molecule has been set to $r_{inter} = 7.5$ Å [8]. Based on this value, we obtain $\sigma_{inter} = 9.88$ Å. The values of ε_{inter} and the cutoff radius c_{inter} are determined by an iterative scheme. The optimal values are considered those giving the best match between the maximum shear strength computed with the two modalities. The best results are obtained for $\varepsilon_{inter} = 106.1$ kcal.mol^{-1} and $c_{inter} =$ 20.0 Å. The optimized parameters of the coarse-grained model are summarized in **Table 1**. The response of the optimized mesoscale model is in very good agreement with the full atomistic model (**Fig. S2**). We also compare strain levels inside the molecule by measuring bond deformation in the backbone of the collagen (**Fig. 1B-C**). The strain distribution matches qualitatively and quantitatively between the two modalities. The collagen molecule shows a stick slip behavior as observed previously between two collagen molecules [16].

2 Discussion

2.1 Choice of the model and location of the mineral in the fibril

During the formation of bone, collagen fibrils are mineralized via the deposition of apatite inside the fibrils. The amount and organization of the mineral inside the fibrils remains controversial. Some studies proposed that after being formed in the gap region of the fibrils, the crystals continue to grow and penetrate into the overlap region of the collagen fibrils [9,17–19]. Others suggested that some, if not most of the crystals, must be present between the fibrils, outside of the fibrillar structure [20–26]. In a recent study, McNally *et al.* used TEM and energy-dispersive X-ray to show that approximately 70% of the mineral forms platelets outside of the fibrils and forms the extra-fibrillar matrix. The remaining mineral was found constrained to the gap zone [6]. In a recent study using full atomistic simulation, we showed that the fibril's gap region was able to accommodate at least 40% mineral [2]. In this study, mineral distribution is based essentially on fibrillar geometry and does not include the mineralization process (growth of the crystals within the overlap region) which cannot be explored in a molecular dynamics timeframe. The mechanical properties at the fibril level are likely to be dependent on the precise arrangement of the interfibrillar crystals [27–29]. However, the mineral distribution obtained in the full atomistic simulation is in good agreement with recent experimental results from Nudelman *et al.* [9]. Therefore, we believe that the organizational uncertainties should not significantly change the mechanisms presented in this study. The influence of mineral distribution in the fibrils will be analyzed in subsequent studies. We focused on finite size fibrils with a diameter of 20 nm since we find that the mechanical behavior reach a plateau as shown for strength and ultimate strain (**Fig. S3**).

2.1 Comparison with experimental data

Due to the extremely small size of mineralized collagen fibril and the complexity in extracting these structures from bone tissue, there are very few experimental results available about their mechanical properties. Hang *et al.* combined AFM and SEM to test in tension individual mineralized collagen fibrils from antler bone, which possesses a structure similar to bone [30]. Since antler bone does not contain extrafibrillar mineral, this allows the study of intrafibrillar mineralization on the mechanics of the fibrils. They found that mineralized collagen fibrils under tension present a linear deformation regime followed by inhomogeneous deformation above a critical strain of 2%. This initial regime matches the toe region in our model corresponding to the alignment of the fibrils along the pulling axis. Our simulation observations match both qualitatively and quantitatively their experimental results and confirm the different regimes and behaviors observed in the experiments (**Fig. S4A**). This supports the hypothesis that stress-strain variations observed experimentally correspond to differences in the mineral content of fibrils. However, the experimental tests show ultimate strength and ultimate strain that are much lower that what we observe *in silico*. It is possible that during the tensile test a pull-out of the fibril from the bone matrix occurs, before the fibril could reach its ultimate mechanical properties.

In a recent study using synchrotron X-rays diffraction, Gupta *et al.* also used antler bone to study mineralized collagen fibrils mechanics [31]. They measured mineral strain with respect to fibrillar strain and showed that mineral strain and stress reach a plateau around 0.13% and 40 MPa respectively. Due to the disorganized shape of the crystals, measuring mineral strain in our simulations proved to be impossible. However, mineral stresses in the simulations present a plateau as had been observed experimentally, confirming that the model is representative of physiological samples.

Mineralized Turkey Leg Tendon (MTLT) has been widely used as a model of mineralized fibrils since its constituting fibrils are arranged in a parallel fashion and its mineral quantity is dependent on the age of the animal. Gupta *et al.* used MTLT to study the effect of mineralization on collagen structure [(32)]. While their study reveals mainly interfibrillar structural effects, the observation are consistent with what we observe at the fibril level. Indeed, the fact that part of the fibrils are relaxed while the remaining keep sustaining the tensile load at the tendon scale can be put in relation to the relaxation of the terminal regions of the molecules during a single fibril tensile test. Moreover, these two phenomena correspond to the transition from linear elastic to non-linear behavior of the samples. Similar to what has been observed in tendons, the inhomogeneous mineral distribution in the fibrils could explain high mechanical properties of mineralized tissues.

2.2 Comparison with previous models

The results presented in this study complete our previous simulation analysis. When compared to the original full atomistic model, the coarse-grained model gives similar trends for the stress-strain curve and gap/overlap length ratio [(2)]. However, the coarse-grained model is significantly stiffer, resulting in low strains. The simulation approach can be partly responsible for the observed differences. Indeed, full atomistic simulations have been performed for only a few equilibrium states with different external pressures applied. This allows for a better relaxation of the structure at the cost of longer simulation time. Using this method to obtain the entire fibril behavior until total failure would have been a prohibitively time consuming approach.
The present study complements our previous analysis of nascent bone using a two-dimensional mesoscale model [(33)]. Here, we develop a larger finite size sample which can capture the diverse deformation mechanisms taking place during the tensile deformation of the fibril until failure. By varying amounts of mineral we were able to explore the role of apatite crystals in collagen's fibril structure.

2.3 Comparison with cross-linked fibrils, single molecule and bone tissue response

Introducing mineral in the fibril allows us to take advantage of every single molecule's mechanical properties. By increasing the interaction forces between the collagen molecules, these molecules form an interconnected network which deforms in a synergistic fashion. As a result, the whole structure's characteristic moduli and transition strain between regime II and III are dictated by the behavior of a single collagen molecule. Still, fibrillar ultimate stress and strain fail to reach the values displayed by a single tropocollagen molecule. The ultimate strain and strength of a fibril remain 20% and 50% lower than that of a single molecule. This discrepancy can be explained by the presence of molecular slippage since the relaxed molecular termini do not contribute to the fibril strength. Besides, mineralization is likely to lead to locally over-constrained molecules which could reach their failure point early on.
Similar to cross-links, the mineral plays the role of a bridging agent that allows the whole structure to take better advantage of a single molecule's properties. The adhesion forces introduced by mineral are weaker than cross-links but reformable since the link is mainly due to electrostatic interactions. These interactions allow a stick-slip deformation process enabling larger energy dissipation compared to cross-linked molecules (**Fig. S4B**). However, bone tissue contains both mineral and cross-links, as shown in figure 8. High mineral density as represented in this study prevents shearing of the fibril, which is the source of deformation of regime III in cross-linked fibrils [(34)]. Similarly, high cross-link density prevent the slippage of molecules' termini, which arise during regime II in non-cross-linked mineralized fibrils. Combining cross-links and mineral therefore have an influence on the third deformation regime, leading to a lower failure strain. (**Fig. S4B**).

When compared to bone tissue experiments performed at the nanoscale, the fibrils present similar stiffness but higher strength [35,36]. Several reason could explain the discrepancy in strength. First, the model focus on a single mineralized collagen fibril. Compared to bone tissue, the model does not contain extrafibrillar mineral which is likely to play an important role on the tissue mechanics [6,37]. Furthermore, the model has been developed for dry interface between collagen and mineral. Adding some water would reduce the adhesion forces between the two components and would favor their sliding and significantly reduce the strength of the system [38]. Finally, the model created here represent a perfect fibril and does not include any defects that are present in biological samples.

2.4 Limitations

The results presented in this study are based on a molecular model of mineralized collagen fibril. There are a few limitations with the use of such models. Quantitatively, our values are between 2 and 3 times larger than experimental results. Entropic effects are a possible explanation for this disagreement since they can lead to a softening of the fibril, particularly in a small-deformation regime. Although we partially take entropic effects into consideration in our model, they might have been minimized through the multi-scale fitting scheme and could be significantly larger in experiments. The size of the model could also partly explain the larger mechanical properties since the fibrils in this study are four to five times smaller than fibrils used experimentally. Indeed, size-dependent effects have been previously shown for collagen fibrils [39]. Large deformation rates used in this study could also be a source of discrepancy. Since the timescale available in molecular modeling is of the order of magnitude of few picoseconds, large strain rates are a necessity to reach large deformation regimes. Such large strain rates have been used in both model fitting and coarse-grained simulation and could be responsible for the model response overestimation. However, a previous study showed that the stress-strain response of a collagen molecule is not significantly altered by loading rate up to 1m/s [14]. Besides, the parameters derived for the model have been extrapolated for quasi-static loading (**Fig. S3D**). Furthermore, no viscosity has been taken into account in the mesoscale model, which make the model less sensitive to time dependent phenomenon. Indeed, the shear strength between collagen and mineral in the coarse-grained framework does not exhibit significant variation for strain rate ranging from 0.01 to 20 m/s (**Fig. S3D**). Therefore, we believe that the deformation mechanisms highlighted here are not significantly altered by the large strain rates employed and we expect the results to be in agreement with observation made experimentally at lower strain rate. To limit the effect of viscosity, we assume that the interface between collagen and mineral does not contain water. This assumption is likely to overestimate the strength of the fibrils. Finally, the models presented here represent flawless fibrils and do not take into account any variability or defect naturally occurring in biological samples. For example, bone mineral contains large amounts of vacancies and substitutions [40] that are likely to affect the mechanics of both the mineral phase and collagen-mineral interaction [41,42]. This is likely to lead to an over-estimation of fibril mechanics. The presence of flaws in biological samples could also explain their early failure compared to our model.

3 References

1. Buehler MJ. Atomistic Elasticity: Linking Atoms and Continuum. In: Buehler MJ, editor. At. Model. Mater. Fail. Boston, MA: Springer US; 2008 [cited 2014 Dec 8]. p. 121–55. Available from: http://link.springer.com/10.1007/978-0-387-76426-9

2. Nair AK, Gautieri A, Chang S-W, Buehler MJ. Molecular mechanics of mineralized collagen fibrils in bone. Nat. Commun. 2013;4:1724.

3. Gautieri A, Vesentini S, Redaelli A, Buehler MJ. Hierarchical structure and nanomechanics of collagen microfibrils from the atomistic scale up. Nano Lett. 2011;11(2):757–66.

4. Orgel JPRO, Irving TC, Miller A, Wess TJ. Microfibrillar structure of type I collagen in situ. Proc. Natl. Acad. Sci. U. S. A. 2006;103(24):9001–5.

5. Lees S, Prostak K, Ingle V, Kjoller K. The loci of mineral in turkey leg tendon as seen by atomic force microscope and electron microscopy. Calcif. Tissue Int. 1994;55:180–9.

6. McNally E a, Schwarcz HP, Botton G a, Arsenault a L. A model for the ultrastructure of bone based on electron microscopy of ion-milled sections. PLoS One. 2012;7(1):e29258.

7. Plimpton S. Fast Parallel Algorithms for Short-Range Molecular Dynamics. J. Comput. Phys. 1995;117(1):1–19.

8. Qin Z, Gautieri A, Nair AK, Inbar H, Buehler MJ. Thickness of hydroxyapatite nanocrystal controls mechanical properties of the collagen-hydroxyapatite interface. Langmuir. 2012;28(4):1982–92.

9. Nudelman F, Pieterse K, George A, Bomans PHH, Friedrich H, Brylka LJ, Hilbers PAJ, de With G, Sommerdijk NAJM. The role of collagen in bone apatite formation in the presence of hydroxyapatite nucleation inhibitors. Nat. Mater. 2010;9(12):1004–9.

10. Astala R, Stott M. First-principles study of hydroxyapatite surfaces and water adsorption. Phys. Rev. B. 2008;78(7):1–11.

11. Brès EF, Hutchison JL. Surface structure study of biological calcium phosphate apatite crystals from human tooth enamel. J. Biomed. Mater. Res. 2002;63(4):433–40.

12. Harding IS, Rashid N, Hing K a. Surface charge and the effect of excess calcium ions on the hydroxyapatite surface. Biomaterials. 2005;26(34):6818–26.

13. Srinivasan M, Uzel SGM, Gautieri A, Keten S, Buehler MJ. Alport syndrome mutations in type IV tropocollagen alter molecular structure and nanomechanical properties. J. Struct. Biol. 2009;168(3):503–10.

14. Gautieri A, Buehler MJ, Redaelli A. Deformation rate controls elasticity and unfolding pathway of single tropocollagen molecules. J. Mech. Behav. Biomed. Mater. 2009;2(2):130–7.

15. Tsai DH. The virial theorem and stress calculation in molecular dynamics. J. Chem. Phys. 1979;70(3):1375.

16. Buehler MJ. Nature designs tough collagen: explaining the nanostructure of collagen fibrils. Proc. Natl. Acad. Sci. U. S. A. 2006;103(33):12285–90.

17. Arsenault AL. Image analysis of collagen-associated mineral distribution in cryogenically prepared turkey leg tendons. Calcif. Tissue Int. 1991;48(1):56–62.

18. Landis WJ, Hodgens KJ, Arena J, Song MJ, McEwen BF. Structural relations between collagen and mineral in bone as determined by high voltage electron microscopic tomography. Microsc. Res. Tech. 1996;33(2):192–202.

19. Silver FH, Landis WJ. Deposition of apatite in mineralizing vertebrate extracellular matrices: A model of possible nucleation sites on type I collagen. Connect. Tissue Res. 2011;52(3):242–54.

20. Eppell SJ, Tong W, Katz JL, Kuhn L, Glimcher MJ. Shape and size of isolated bone mineralites measured using atomic force microscopy. J. Orthop. Res. 2001;19(6):1027–34.

21. Su X, Sun K, Cui F., Landis W. Organization of apatite crystals in human woven bone. Bone. 2003;32(2):150–62.

22. Landis WJ, Hodgens KJ, Song MJ, Arena J, Kiyonaga S, Marko M, Owen C, McEwen BF. Mineralization of collagen may occur on fibril surfaces: evidence from conventional and high-voltage electron microscopy and three-dimensional imaging. J. Struct. Biol. 1996;117(1):24–35.

23. Alexander B, Daulton TL, Genin GM, Lipner J, Pasteris JD, Wopenka B, Thomopoulos S. The nanometre-scale physiology of bone: steric modelling and scanning transmission electron microscopy of collagen-mineral structure. J. R. Soc. Interface. 2012;9(73):1774–86.

24. Pidaparti R, Chandran A. Bone mineral lies mainly outside collagen fibrils: predictions of a composite model for osternal bone. J. Biomech. 1996;29(7):909–16.

25. Bonar LC, Lees S, Mook H a. Neutron diffraction studies of collagen in fully mineralized bone. J. Mol. Biol. 1985;181(2):265–70.

26. Balooch M, Habelitz S, Kinney JH, Marshall SJ, Marshall GW. Mechanical properties of mineralized collagen fibrils as influenced by demineralization. J. Struct. Biol. 2008;162(3):404–10.

27. Weiner S, Wagner HD. The material bone: Structure mechanical function relations. Annu. Rev. Mater. Sci. 1998;28:271–98.

28. Rho JY, Kuhn-Spearing L, Zioupos P. Mechanical properties and the hierarchical structure of bone. Med. Eng. Phys. 1998;20(2):92–102.

29. Jäger I, Fratzl P. Mineralized collagen fibrils: a mechanical model with a staggered arrangement of mineral particles. Biophys. J. 2000;79(4):1737–46.

30. Hang F, Barber AH. Nano-mechanical properties of individual mineralized collagen fibrils from bone tissue. J. R. Soc. Interface. 2011;8(57):500–5.

31. Gupta HS, Krauss S, Kerschnitzki M, Karunaratne A, Dunlop JWC, Barber a H, Boesecke P, Funari SS, Fratzl P. Intrafibrillar plasticity through mineral/collagen sliding is the dominant mechanism for the extreme toughness of antler bone. J. Mech. Behav. Biomed. Mater. 2013;1–17.

32. Gupta H, Messmer P, Roschger P, Bernstorff S, Klaushofer K, Fratzl P. Synchrotron Diffraction Study of Deformation Mechanisms in Mineralized Tendon. Phys. Rev. Lett. 2004;93(15):158101.

33. Buehler MJ. Molecular nanomechanics of nascent bone: fibrillar toughening by mineralization. Nanotechnology. 2007;18(29):295102.

34. Depalle B, Qin Z, Shefelbine SJ, Buehler MJ. Influence of cross-link structure, density and mechanical properties in the mesoscale deformation mechanisms of collagen fibrils. J. Mech. Behav. Biomed. Mater. 2014;1–13.

35. Schwiedrzik J, Raghavan R, Bürki A, LeNader V, Wolfram U, Michler J, Zysset P. In situ micropillar compression reveals superior strength and ductility but an absence of damage in lamellar bone. Nat. Mater. 2014;13(7):740–7.

36. Luczynski KW, Steiger-Thirsfeld A, Bernardi J, Eberhardsteiner J, Hellmich C. Extracellular bone matrix exhibits hardening elastoplasticity and more than double cortical strength: Evidence from homogeneous compression of non-tapered single micron-sized pillars welded to a rigid substrate. J. Mech. Behav. Biomed. Mater. 2015;1–12.

37. Hellmich C, Barthélémy J-F, Dormieux L. Mineral–collagen interactions in elasticity of bone ultrastructure – a continuum micromechanics approach. Eur. J. Mech. - A/Solids. 2004;23(5):783–810.

38. Eberhardsteiner L, Hellmich C, Scheiner S. Layered water in crystal interfaces as source for bone viscoelasticity: arguments from a multiscale approach. Comput. Methods Biomech. Biomed. Engin. 2012;(February 2015):1–16.

39. Shen ZL, Dodge MR, Kahn H, Ballarini R, Eppell SJ. Stress-strain experiments on individual collagen fibrils. Biophys. J. 2008;95(8):3956–63.

40. Cazalbou S, Combes C, Eichert D, Rey C. Adaptative physico-chemistry of bio-related calcium phosphates. J. Mater. Chem. 2004;14(14):2148.

41. Ren F, Lu X, Leng Y. Ab initio simulation on the crystal structure and elastic properties of carbonated apatite. J. Mech. Behav. Biomed. Mater. 2013;26:59–67.

42. Sun JP, Song Y, Wen GW, Wang Y, Yang R. Softening of hydroxyapatite by vacancies: a first principles investigation. Mater. Sci. Eng. C. Mater. Biol. Appl. 2013;33(3):1109–15.

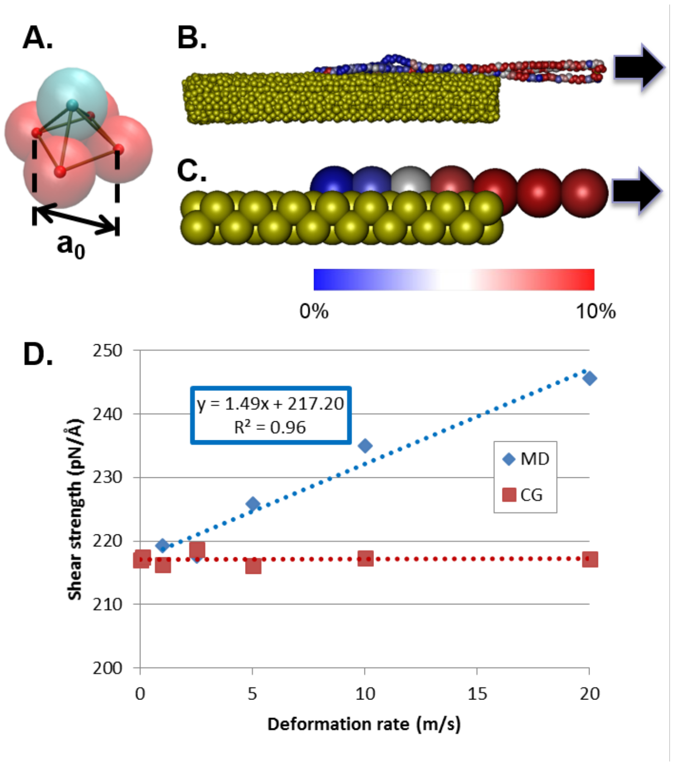

Figure S1: (A) Geometry of the collagen-mineral interaction in the coarse-grained model. The 4 red beads are representing hydroxyapatite crystal and the blue bead represents a segment of a collagen molecule. (B) Full atomistic model of the collagen-hydroxyapatite adhesion and (C) corresponding coarse-grained model. For clarity, only the backbone of the collagen molecule is represented. The color scale represents the strain in the molecule. The coarse-grained model presents a similar behavior and strain distribution as seen in the full-atomistic simualtion. (D) Evolution of the maximum shear strength measure using full atomistic simulation (MD) and the coarse-grained representation (CG). The extrapolated value of the shear strength for a quasi-static deformation (0 m/s) is used to fit the mesoscale model. Since no viscosity has been included, the mesoscale model does not exhibit strain rate dependence.

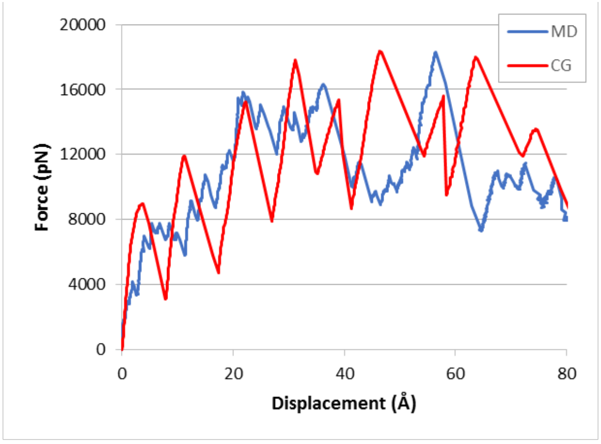

Figure S2: Force-displacement response for the collagen-hydroxyapatite adhesion computed with a full atomistic simulation (blue curve) and with optimized coarse-grained model developed in this study (red curve). Both full atomistic and mesoscale model present a saw-tooth behavior characteristic of the stick slip behavior of collagen.

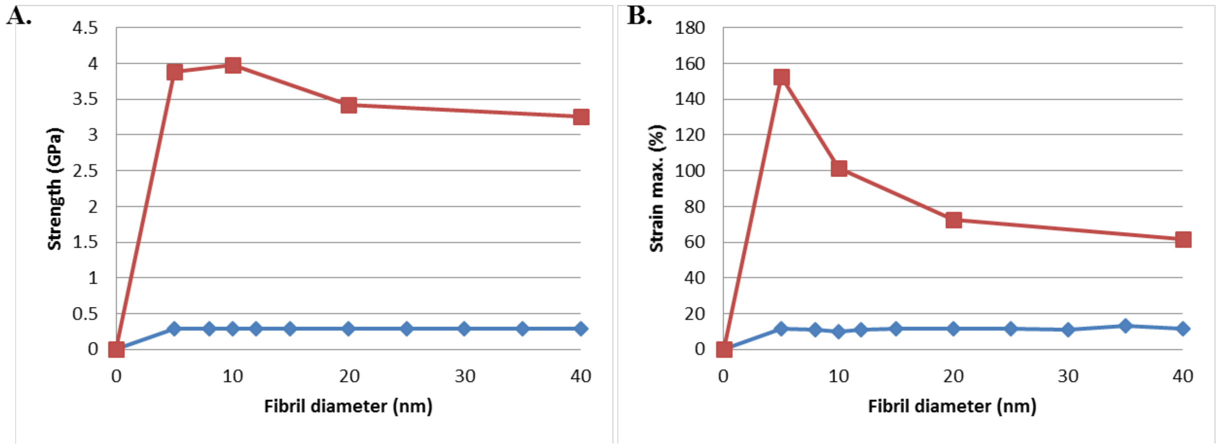

Figure S3: Evolution of mechanical properties of the fibrils as a function of fibril diameter. (graph for non-mineralized fibrils and 25% of mineral)

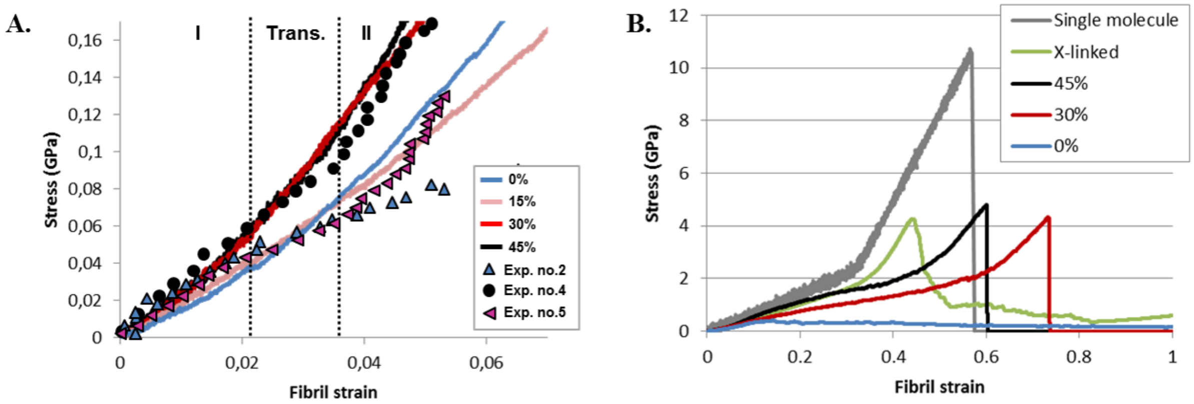

Fig. S4: (A) Comparison between the model of mineralized collagen fibrils response and experimental results for a tensile test reproduced from ref. [30]. **(B)** Comparison of stress-strain curves from collagen fibrils containing 0, 30 and 45% mineral with the response of single collagen molecules and fully cross-linked fibril (2 cross-links per molecules) reproduced from one of our previous study [34].
